# A Size and Boundary Effects Model for Quasi-Brittle Fracture

**DOI:** 10.3390/ma9121030

**Published:** 2016-12-21

**Authors:** Xiaofeng Gao, Georg Koval, Cyrille Chazallon

**Affiliations:** ICUBE Laboratory, CNRS, National Institute of Applied Sciences of Strasbourg, 24 Boulevard de la Victoire, Strasbourg 67084, France; xiaofeng.gao@insa-strasbourg.fr (X.G.); cyrille.chazallon@insa-strasbourg.fr (C.C.)

**Keywords:** nominal strength, size effect, boundary effect, failure model, quasi-brittle

## Abstract

The fracture behaviors of quasi-brittle materials are commonly specimen size (size effect) and crack size (boundary effect) dependent. In this study, a new failure model is developed for characterizing the size and boundary effects. The derivative of the energy release rate is firstly introduced to predict the nominal strength dominated by the strength mechanism. Combined with the energy criterion for the energy mechanism, an asymptotic model is developed to capture the effect of any crack size on the nominal strength, and its expression for geometrically similar specimens is also established, which is able to characterize the size effect. Detailed comparisons of the proposed model with the size effect law and the boundary effect model are performed, respectively. The nominal strength predictions based on the proposed model are validated with the experimental results of cracked three-point bending beam specimens made of concrete, of limestone and of hardened cement paste and compared with the model predictions given by the size effect law and the boundary effect model.

## 1. Introduction

Nominal strengths of quasi-brittle materials, like concretes, rocks, some types of ceramics, etc., are commonly specimen size (size effect) [1,2,3,4] and crack size dependent (boundary effect) [5,6,7,8]. The phenomena exist due to the fact that there is a micro-crack region called the Fracture Process Zone (FPZ) around the tip of the defect. The fracture behavior starts to be size dependent when the FPZ is relatively large relative to the specimen size, while for sufficiently small FPZ with respect to the size of the specimen, the failure prediction can be easily achieved by the Linear Elastic Fracture Mechanics (LEFM). On the other hand, the boundary effect is determined by the size of a fully-developed FPZ, its distance to the front boundary measured by the crack length and its distance to the back boundary measured by the un-cracked ligament. When the crack length or un-cracked ligament is smaller than a certain size, the fracture behavior is affected by the specimen boundaries [7]. Quasi-brittle materials commonly have relatively large values of FPZ. Therefore, it is important to have a better understanding of the size and boundary effects of quasi-brittle materials.

In order to characterize the size and boundary effects induced by the specimen size and crack size, several elastic stress field-based models, fracture mechanics-based models, combined stress and energy models and asymptotic approaches have been established by researchers. The first three models are classified as Theories of Critical Distance (TCDs), because they are associated with a length scale usually proportional to Irwin’s characteristic length $l_{\mathrm{ch}}$ [9], which reads:

(1) $$l_{\mathrm{ch}}=\frac{G_{c}E}{f_{t}^{2}}=\frac{K_{c}^{2}}{f_{t}^{2}}$$

where $G_{c}$ is the material fracture energy, *E* is Young’s modulus, $K_{c}$ is the fracture toughness and $f_{t}$ is the material tensile strength.

Based on the stress criterion, the point and line stress methods state that crack propagation occurs when circumferential stress at some critical distance from the crack tip reaches a given critical value [10,11,12,13]. The fracture mechanics-based models, such as the imaginary crack model [14,15], finite fracture mechanics [16] and the volume-based strain energy density criterion [13,17], use only the energy or energy density for the failure analysis. The combined stress and energy models [18,19] assume that failure occurs when both criteria are fulfilled. The length scale in these models usually depends on the properties of the material and proportional to Irwin’s characteristic length $l_{\mathrm{ch}}$. However, for quasi-brittle materials, the value of the characteristic length scale may become too large when compared to the specimen size, which makes the direct implementation of these approaches impossible [12].

In terms of the asymptotic approaches, such as Hu–Duan’s boundary effect model, and Bažant’s size effect law, all are able to characterize the size and boundary effects induced by the crack size and specimen size. The size effect models emphasize the influence of the physical sample size on the nominal strength, and the crack length dependence of the fracture properties is not emphasized. The most commonly-known specimen size-based model is the Size Effect Law (SEL) proposed by Bažant [1,2,3,4,20]. SEL is defined by asymptotically matching the extreme responses of geometrically similar specimens of different sizes. It was initially developed for cracked specimens (Type 2 SEL) and has been extended to un-cracked specimens (Type 1 SEL). The other models proposed by Carpinteri and Chiaia [21,22], Carpinteri et al. [23] and Karihaloo [24] are also able to characterize effectively the transition of quasi-brittle failure from the maximum tensile strength criterion to the LEFM criterion for geometrically similar specimens. Each size effect model has at least two experimental parameters that can be adjusted to fit the experimental results.

The boundary effect model is based on a hypothesis about the effect of crack length on the nominal strength. According to boundary effect and using an equivalent crack length, Hu and Duan [6,7] proposed a Boundary Effect Model (BEM) different from the specimen size-based models, which can be used to predict the nominal strengths of a finite or infinite width specimen containing different crack sizes. The boundary effect model is not restricted to geometrically similar specimens, and it proves that the boundary effect induced by the crack length can exist even in large specimens. Based on this point, Hu and Duan concluded the common size effect associated with geometrically similar specimens is only a special case of the boundary effect [7]. However, this model was critically examined by Yu et al. [25]. Significant theoretical objections were raised, and the further experimental verification [26] proved that BEM is distinctly inferior to the Type 1 SEL and Type 2 SEL. In the later improved model of the Universal Size Effect Law (USEL), Bažant and Yu [27] believe that the dependence of the nominal strength of structure on the crack length at constant specimen size is a special case of the USEL, and USEL is more realistic than the boundary effect model.

In this study, a new size and boundary effect model is developed for characterizing the fracture dependence on the specimen size and crack size. The derivative of energy release rate $G^{\prime }$ is firstly introduced to give the nominal strength prediction by the strength mechanism. Combined with the LEFM criterion that predicts the nominal strength based on the energy mechanism, an asymptotic model is developed to capture the full process of crack initiation and crack propagation. In Section 3, a geometrical correction factor H(α) for the derivative of energy release rate $G^{\prime }$ is defined, and then, the expressions for the derivative of energy release rate $G^{\prime }$ and equivalent crack length $a_{e}$ are derived. Based on this information, the proposed model is established and compared with the boundary effect model and the Type 2 size effect law. In Section 4, the nominal strength predictions based on the proposed model are compared with the experimental results of Cracked Three-Point Bending (C-TPB) beam specimens made of concrete, of limestone and of hardened cement paste and the model predictions of the Type 2 size effect law and boundary effect model.

## 2. Size and Boundary Effects

### 2.1. Size Effect Induced by Specimen Sizes

**Figure 1:**
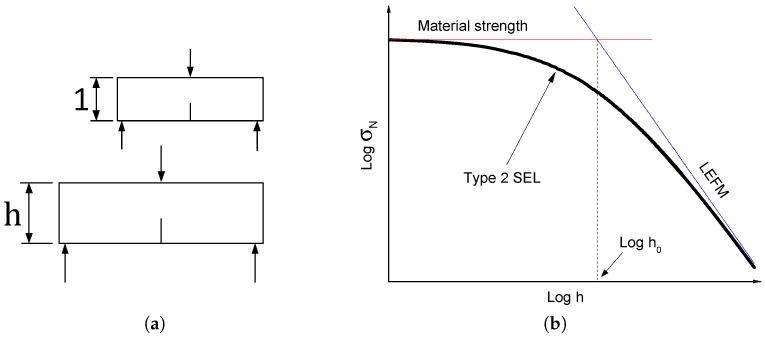
(**a**) Similar cracked structures and (**b**) Size Effect Law (SEL) bridging the failure mechanisms of material strength and Linear Elastic Fracture Mechanics (LEFM).

Quasi-brittle materials obey on a small scale the strength theory, characterized by material strength $f_{t}$, and on a large scale the LEFM, characterized by toughness $G_{c}$. The combination of $f_{t}$ and $G_{c}$ yields Irwin’s characteristic length $l_{\mathrm{ch}}=G_{c}E/f_{t}^{2}$ [9] and separates the small and large scales. Based on an approximate energy release analysis, SEL was derived for geometrically similar specimens (Figure 1a) in 1984 [1] and reformulated in 1991 [4]. For Type 2 failures, which are occurring when there is a notch or a large stress-free crack formed before reaching the maximum loading, the law reads:

(2) $$\sigma_{{}_{N}}=\hat{B}f_{t}{\left(1+\frac{h}{h_{0}}\right)}^{-1/2}$$

where $\hat{B}$ is a positive dimensionless constant depending on the geometry of the structure; $f_{t}$ is the material tensile strength; $h_{0}$ is a constant proportional to Irwin’s characteristic length $l_{\mathrm{ch}}$, at which the failure laws based on material strength and LEFM intersect, as shown in Figure 1b. $h_{0}$ and $\hat{B}$ characterize the structure geometry.

Since Type 2 SEL is not valid when the crack to height ratio α tends to zero, the Type 1 SEL [28] was proposed after Type 2 SEL and applied to structures failing at crack initiation from a smooth surface. The Type 1 SEL reads [26]:

(3) $$\sigma_{N}=f_{r,\infty }{\left(1+\frac{rh_{b}}{h+l_{p}}\right)}^{1/r}$$

where $f_{r,\infty }$, $h_{b}$, $l_{p}$ and *r* are constants of the model whose values need to be determined empirically.

In order to describe the continuous transition between these two types of size effects, the Universal Size Effect Law (USEL) was firstly defined by Bažant [29] and then improved by Bažant and Yu [27]. USEL has been validated with various experimental results and shown to fit the test results quite well [30].

### 2.2. Boundary Effect Induced by Crack Sizes

**Figure 2:**
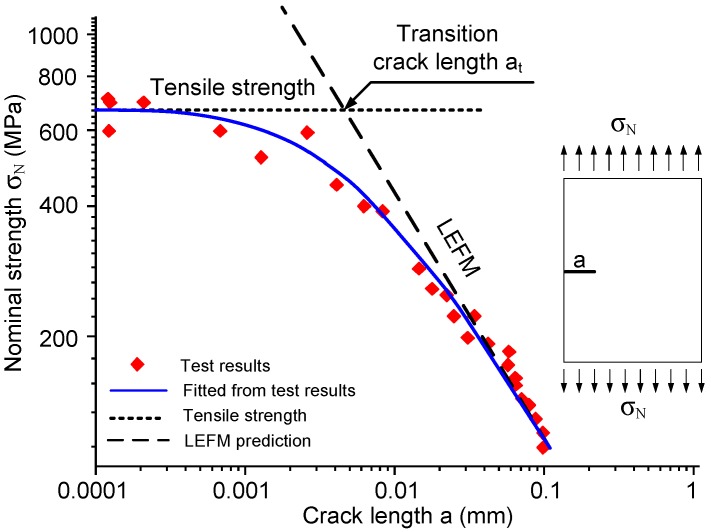
Nominal strengths versus crack length in SiC (modified from [12]).

Figure 2 shows typical test results measuring the nominal strength of a specimen of Silicon Carbide (SiC) containing different crack sizes [12]. The energy criterion of LEFM works for sufficiently large cracks, while the tensile strength seems to be the failure stress when the crack length is below 0.001 mm. Between these two conditions, both the stress criterion and energy criterion are not applicable. The stress criterion would provide a null strength due to the stress singularity at the crack tip, while the energy criterion would give an unreal nominal strength, which is higher than the material tensile strength. The test results shown in Figure 2 indicate that the nominal strength transits smoothly from the tensile strength to LEFM. The intersection of the LEFM line and the line corresponding to the tensile strength is defined as the transition crack length, which can be calculated by the following expression [7]:

(4) $$a_{t}=\frac{G_{c}E}{{\left(1.12f_{t}\right)}^{2}\pi }=\frac{l_{\mathrm{ch}}}{1.12^{2}\pi }$$

The transition crack length $a_{t}$ is proportional to Irwin’s characteristic length $l_{\mathrm{ch}}$. Therefore, it is also a material-dependent parameter relating to toughness $G_{c}$ and tensile strength $f_{t}$. It should be noticed that SiC is a brittle material with a small value of $a_{t}$. Quasi-brittle materials commonly have larger values of $a_{t}$, which can be around 40 mm for concrete material. The example of SiC is taken here to illustrate the influence of crack size on the nominal strength, which are the same for brittle and quasi-brittle materials.

## 3. Proposed Failure Model

It is known that the local stress criterion and energy criterion are unable to predict the failure of a specimen containing relatively small or intermediate cracks [7,12]. In this section, the derivative of energy release rate $G^{\prime }$ is introduced to predict the nominal strength given by the strength mechanism. On the other hand, the energy criterion is sufficient to give a good prediction for the material rupture dominated by the energy mechanism. Based on the derivative of the energy release rate and energy criterion, the asymptotic model is found to cover the nominal strength prediction for any crack size. The proposed model is established based on the Cracked Three-Point Bending (C-TPB) beam specimen, but can be easily generalized to many other structures and different boundary conditions, such as a center or edge cracked plate, a cracked pure bending specimen, a compact tension test specimen, etc.

### 3.1. Derivative of the Energy Release Rate

**Figure 3:**
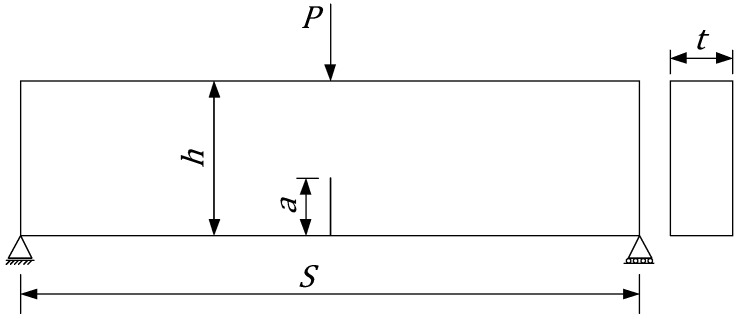
Cracked Three-Point Bending (C-TPB) beam specimen.

Consider a C-TPB beam (Figure 3) with a crack to height ratio α=a/h. The energy release rate *G* can be written as [31]:

(5) $$G=\frac{{\left[A\left(\alpha \right)\sigma \right]}^{2}\pi a}{E}$$

where $\sigma =\left(3PS\right)/\left(2h^{2}t\right)$; A(α) is the geometrical correction factor for the energy release rate; *a* is the crack length; *h*, *S* and *t* are the height, span and thickness of the beam, respectively; *E* is the Young’s modulus of the material. The geometrical correction factor A(α) can be found numerically or analytically. The empirical formulas for different structures have been derived by many researchers [31]. For the beam with a span to height ratio S/h=4, Equation (6) gives 0.5% accuracy of *G* for any crack to height ratio α,

(6) $$A\left(\alpha \right)=\frac{1}{\sqrt{\pi }}\frac{1.99-\alpha \left(1-\alpha \right)\left(2.15-3.93\alpha +2.7\alpha^{2}\right)}{\left(1+2\alpha \right){\left(1-\alpha \right)}^{3/2}}$$

The product rule is used to find the derivative of the energy release rate with respect to the crack length *a*. In Equation (5), $\sigma^{2}\pi /E$ is a constant, and the derivative of $A^{2}\left(\alpha \right)a$ equals $A^{2}\left(\alpha \right)+2A\left(\alpha \right)A^{\prime }\left(\alpha \right)\alpha^{\prime }a$, with $\alpha^{\prime }=1/h$ ($\alpha^{\prime }a=\alpha$). Therefore, the derivative of the energy release rate with respect to the crack length *a* can be written as follows:

(7) $$G^{\prime }=\frac{{\left[H\left(\alpha \right)\sigma \right]}^{2}\pi }{E}$$

where H(α) is defined as the correction factor for the derivative of energy release rate $G^{\prime }$, which reads:

(8) $$H\left(\alpha \right)=\sqrt{A^{2}\left(\alpha \right)+2A\left(\alpha \right)\times dA\left(\alpha \right)/d\alpha \times \alpha }$$

**Figure 4:**
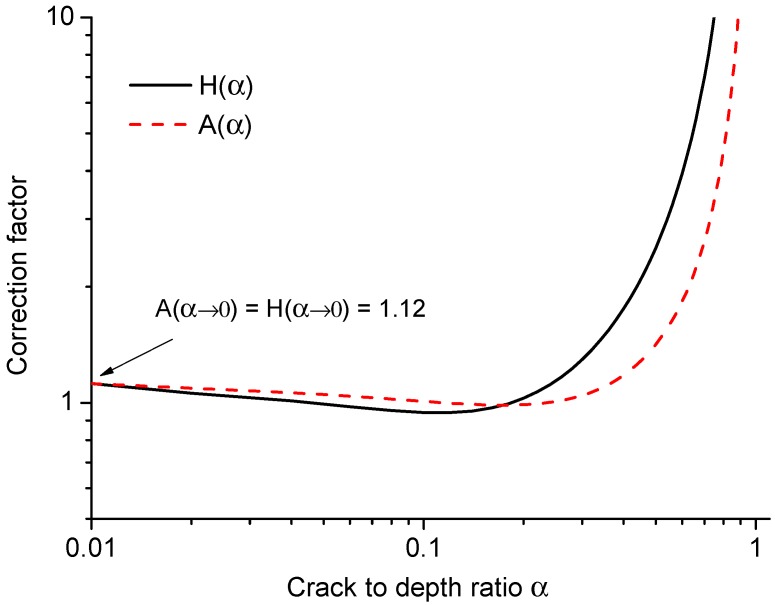
Correction factors A(α) and H(α) versus the crack to height ratio α.

Figure 4 shows the values of two correction factors A(α) and H(α) for *G* and $G^{\prime }$ with respect to the crack to height ratio α. When α<0.01, the difference of the two factors is very small, and they all tend to infinity when α→1.

The derivative of the energy release rate, as shown in Equation (7), is proportional to the geometrically corrected stress value H(α)σ, where σ is obtained as the stress at the bottom of the mid-span without considering the crack; H(α) can be regarded also as a correction factor, which can take the crack into consideration. The critical value of the derivative of energy release rate $G_{c}^{\prime }$ is obtained when $\sigma_{0}\to f_{t}$ and $a_{0}\to 0$. For the C-TPB specimen with a span to height ratio equal to four, $G_{c}^{\prime }$ can be calculated by Equations (6) and (7), as shown in Equation (9). $G_{c}^{\prime }$ is a material constant related to the tensile strength $f_{t}$ and Young’s modulus *E*. In other words, the critical value for the corrected stress H(α)σ is $1.12f_{t}$.

(9) $$G_{c}^{\prime }=\frac{{\left(1.99f_{t}/\sqrt{\pi }\right)}^{2}\pi }{E}\approx \frac{{\left(1.12f_{t}\right)}^{2}\pi }{E}$$

By relating Equations (7) and (9), the nominal strength $\sigma_{N}$ based on the derivative of energy release rate $G^{\prime }$ can be calculated by the following expression:

(10) $$\sigma_{N}=\frac{1.12f_{t}}{H(\alpha )}$$

or one can simply consider that the failure is reached when the corrected stress value $H\left(\alpha \right)\sigma =1.12f_{t}$. Hence, the nominal strength $\sigma_{N}$ of the strength mechanism can be predicted by Equation (10). When α<0.01, as shown in Figure 4, H(α)≈1.12; therefore, $\sigma_{N}\approx f_{t}$. It should be noted that H(α) has different expressions for different structures, which is related to A(α), and can be found analytically or numerically. The same as the boundary effect model, Equation (10) can capture the boundary effect when the distance from the fracture process zone to the lower boundary (crack length) is small; the rupture behavior is mainly dominated by the strength mechanism. It should also be pointed out that Equation (10) is not able to give the nominal strength predictions for intermediate cracks, because H(α) experiences a slight decrease before it increases towards infinity. This would lead to the unreal nominal strength predictions being higher than the material tensile strength by simply using Equation (10) for intermediate crack sizes.

### 3.2. Asymptotic Model

For a relatively large crack in a large specimen, the material rupture is dominated by the energy criterion. By taking the definition of H(α) into account, the expression for the energy release rate shown in Equation (5) can be written as follows:

(11) $$G=\frac{{\left[A\left(\alpha \right)\sigma H\left(\alpha \right)/H\left(\alpha \right)\right]}^{2}\pi a}{E}=\frac{{\left[H\left(\alpha \right)\sigma \right]}^{2}\pi a_{e}}{E}$$

where:

(12) $$a_{e}=\frac{A^{2}\left(\alpha \right)}{H^{2}\left(\alpha \right)}a$$

**Figure 5:**
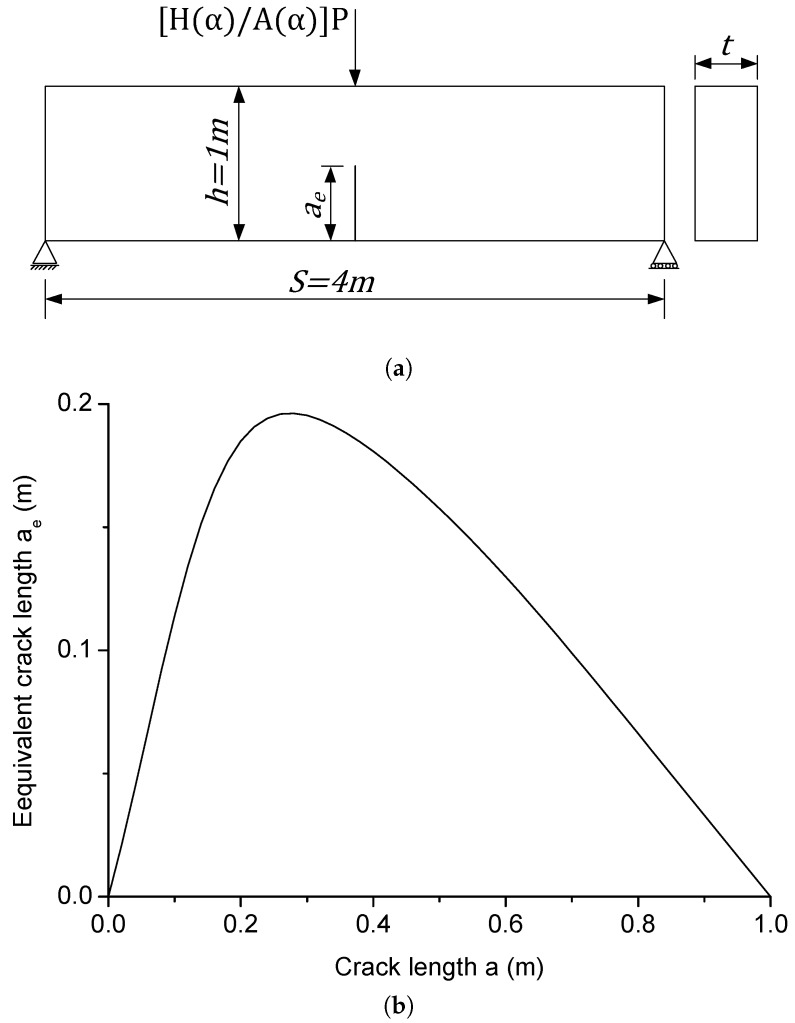
(**a**) Beam with equivalent loading and equivalent crack length $a_{e}$ and (**b**) equivalent crack length $a_{e}$ versus real crack length *a*.

$a_{e}$ is defined as the equivalent crack length, which depends on the initial crack length *a* and the crack to height ratio α. After introducing the concept of equivalent crack length $a_{e}$, the cracked beam specimen with initial crack length *a* and loading *P* is equivalent to the beam with crack length $a_{e}$ and loading [H(α)/A(α)]P (see Figure 5a). Hence, the energy release rate at the tip of the equivalent crack is the same as the value of the initial crack. Figure 5b illustrates the variation of equivalent crack length $a_{e}$ with respect to real crack length *a* for the beam specimen with h=1 m and S=4 m. $a_{e}$ tends to zero when α→0 and α→1.

The critical energy release rate is the material toughness $G_{c}$; along with the definition of transition crack length $a_{t}$ shown in Equation (4), the nominal strength given by the energy criterion can be written as Equation (13).

(13) $$\sigma_{N}=\frac{1.12f_{t}}{H(\alpha )}{\left(\frac{a_{e}}{a_{t}}\right)}^{-1/2}$$

It is noticed that Equations (10) and (13) can be bridged together to predict the material rupture from the strength mechanism to the energy mechanism; thus, an asymptotic model is developed, as shown in Equation (14):

(14) $$\sigma_{N}=\frac{1.12f_{t}}{H(\alpha )}{\left(1+\frac{a_{e}}{a_{t}}\right)}^{-1/2}$$

Same as the Hu–Duan boundary effect model [7], Equation (14) is also a crack size-based model, which estimates the effect of crack length on the nominal strength. Despite the similarity in their shapes, the definitions and expressions of the equivalent crack length and the geometrical correction factor in Equation (14) are fundamentally different from those in the boundary effect model. The differences between the proposed model and the Hu–Duan boundary effect model has been presented in Appendix A.

**Figure 6:**
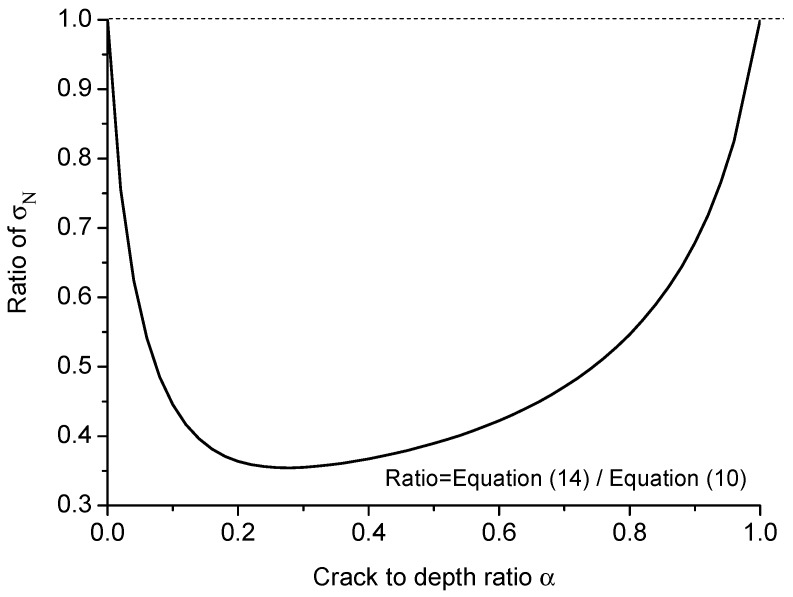
Ratio of nominal strength $\sigma_{N}$ given by Equations (14) and (10) versus the crack to height ratio α.

It is interesting to notice that the ratio of nominal strength $\sigma_{N}$ given by Equations (14) and (10) tends to one for very small and very large crack to height ratios α, as shown in Figure 6. This phenomenon indicates that for these two extreme cases, the derivative of energy release rate $G^{\prime }$ is the dominant factor for the rupture. Take the beam shown in Figure 5a for instance; when the real crack length approaches one, on the contrary, the equivalent crack length $a_{e}$ tends to zero; hence, the contribution of the energy release rate part in Equation (14) on the nominal strength $\sigma_{N}$ becomes much weaker than the derivative of energy release rate $G^{\prime }$ and can be eventually neglected at a certain moment.

**Figure 7:**
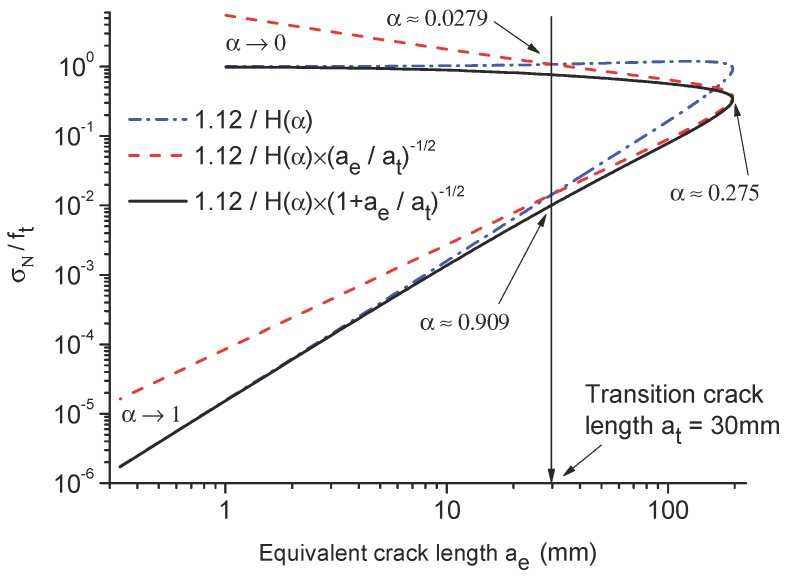
Nominal strength to tensile strength ratios versus equivalent crack length $a_{e}$.

Figure 7 presents the nominal strength to tensile strength ratios of the cracked beam structure versus equivalent crack length $a_{e}$ (beam height *h* = 1 m). When α<0.0279 and α>0.909, the rupture behavior is mainly dominated by the strength mechanism (derivative of the energy release rate), which has already been discussed. This phenomenon can be explained as the boundary effect [7], because the distances of the fracture process zone to the lower boundary measured by the crack length (α<0.0279) and to the upper boundary measured by the un-cracked ligament (α>0.909) are too small; therefore, the fracture behaviors are influenced by the specimen boundaries and dominated by the strength mechanism. When equivalent crack length $a_{e}$ is bigger than the transition crack length $a_{t}$, the rupture behavior is mainly dominated by the energy mechanism. α=0.275 provides the strongest contribution of the strength mechanism on the failure of the beam.

**Figure 8:**
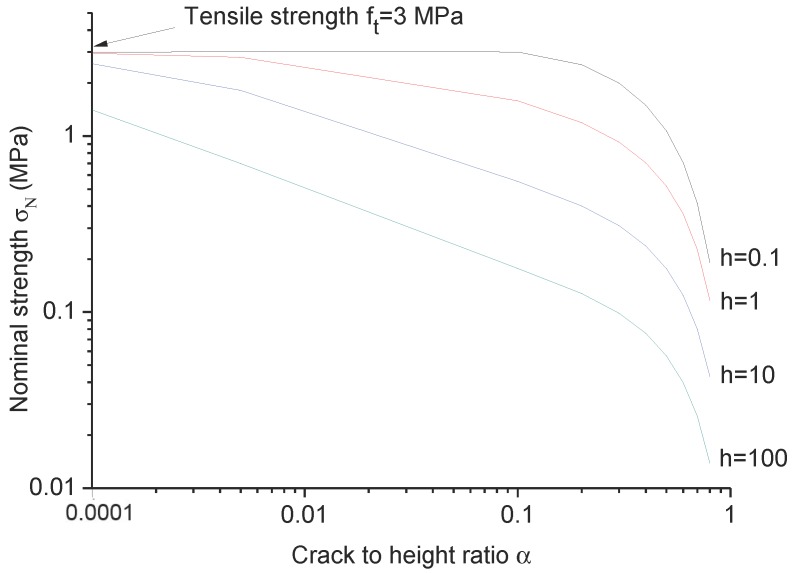
Nominal strengths versus the crack to height ratio α for various beam heights.

The asymptotic model shown in Equation (14) can be used to predict the failure stress for any crack size (or any crack to height ratio α), which is able to give a smooth transition from small, intermediate cracks to large cracks. Figure 8 presents an example of the nominal strength versus crack to height ratio α for beam height *h* = 0.1 m, 1 m, 10 m, 100 m. By using Equation (14) and the information of the material parameters, including tensile strength $f_{t}$ = 3.0 MPa, fracture toughness $K_{c}=\sqrt{G_{c}E}=1.0\;\mathrm{MPa}\cdot m^{1/2}$ and transition crack length $a_{t}$ = 28.2 mm, the nominal strengths can be easily predicted; they are plotted in Figure 8. For beam height *h* = 100 m, a smaller crack to height ratio α is needed to have the nominal strength approximately equal to the tensile strength $f_{t}$.

Due to the size effects, the material parameters may vary for different specimen shapes and sizes. Analogous to SEL, the material and geometrical information, including $H\left(\alpha \right)/\left(1.12f_{t}\right)$ and $a_{t}$, can also be identified from the test results (geometrically similar tests) if they are not sufficient or not easy to calculate. By setting $Y=1/(\sigma_{N}^{2})$, Equation (14) gives a linear regression plot Y=JX+C, shown in Equation (15), from which *J* and *C* can be identified as the slope and intercept.

(15) $$Y=\frac{1}{\sigma_{N}^{2}}={\left[\frac{H(\alpha )}{1.12f_{t}}\right]}^{2}\frac{a_{e}}{a_{t}}+{\left[\frac{H(\alpha )}{1.12f_{t}}\right]}^{2}$$

with $a_{e}=X$, ${\left[H\left(\alpha \right)/\left(1.12f_{t}\right)\right]}^{2}/a_{t}=J$, ${\left[H\left(\alpha \right)/\left(1.12f_{t}\right)\right]}^{2}=C$.

It should be pointed out that *J* and *C* are constants only for geometrically similar specimens (same crack to height ratio α), due to the identical correction factor H(α).

### 3.3. Proposed Failure Model for Geometrically Similar Specimens

The asymptotic model in Equation (14) can predict the nominal strength for different crack sizes and has a similar shape as the Type 2 SEL shown in Equation (2). Since $a_{0}=\alpha h$, the equivalent crack length $a_{e}$ in Equation (14) can be replaced by $a_{e}={\left[A\left(\alpha \right)/H\left(\alpha \right)\right]}^{2}\alpha h$. Then, a transition beam height $h_{t}$ can be defined as:

(16) $$h_{t}=\frac{a_{t}}{\alpha }{\left[\frac{H(\alpha )}{A(\alpha )}\right]}^{2}$$

$h_{t}$ is a function of transition crack length $a_{t}$ and the crack to height ratio α, which is proportional to the characteristic length $l_{\mathrm{ch}}$. Hence, the asymptotic model of the proposed model for geometrically similar specimens is developed, which reads:

(17) $$\sigma_{N}=\frac{1.12f_{t}}{H(\alpha )}{\left(1+\frac{h}{h_{t}}\right)}^{-1/2}$$

Equation (17) has the same shape as Type 2 SEL. $h_{t}$ and $h_{0}$ are the transitional sizes where the material strength and LEFM intersect and are all proportional to Irwin’s characteristic length $l_{\mathrm{ch}}$. $\hat{B}$ in Equation (2) is a positive dimensionless constant depending on the geometry of the structure, the same as 1.12/H(α) in Equation (17). Therefore, the proposed model is able to predict the rupture of geometrically similar specimens, the same as Type 2 SEL. A detailed comparison of the proposed model and Type 2 SEL is presented in Appendix B, proving that when the length scale parameter is the same, the proposed failure model shown in Equations (14) and (17) and Type 2 SEL are identical, although the proposed model is presented in two different forms.

**Figure 9:**
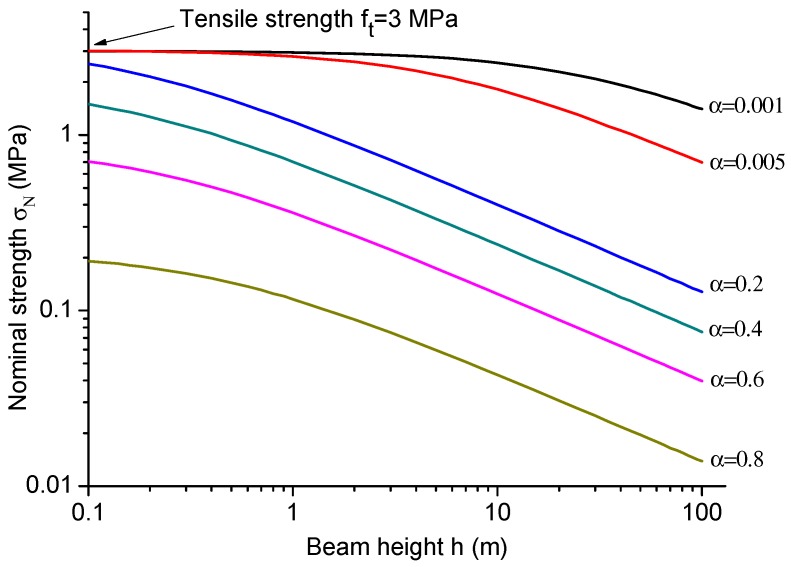
Nominal strengths versus beam height for various crack to height ratios.

Figure 9 presents an example of the nominal strengths with respect to beam heights for various crack to height ratios. The material parameters are identical as the parameters adopted in Figure 8. By using Equation (14) or (17), the nominal strength can be predicted for both geometrically similar specimens (Figure 9) and a certain beam size with various crack to height ratios α (Figure 8). However, not like the Type 1 SEL, the proposed model of Equations (14) and (17) cannot predict the size effect of crack initiation from the free surface, because it will give the same nominal strength for different specimen sizes.

## 4. Model Validations

### 4.1. Concrete Experiments

Bažant et al. [32,33] carried out a series of experiments with a C-TPB specimen with a similar geometry, to investigate the size effects in concrete specimens. The eight specimens have the same crack to height ratio α=0.33, the same span to height ratio S/h=4 and a fixed thickness *t* = 25.4 mm. The concrete presents the following mechanical properties: average tensile strength $f_{t}$ = 3.0 MPa and fracture toughness $K_{c}=\sqrt{G_{c}E}=1.23\;\mathrm{MPa}\cdot m^{1/2}$. The corresponding transition crack length $a_{t}$ = 42.66 mm. The two correction factors A(α) and H(α) are 1.08 and 1.42, respectively, and then, the equivalent crack length $a_{e}$ for each beam can be calculated accordingly.

The beam dimensions, equivalent crack length $a_{e}$, failure loads $P_{\max}$ and the nominal strengths $\sigma_{N}$ for the eight specimen are listed in Table 1. The nominal strengths $\sigma_{N}$ can be written for the C-TPB specimen as:

(18) $$\sigma_{N}=\frac{3P_{\max}S}{2h^{2}t}$$

**Figure 10:**
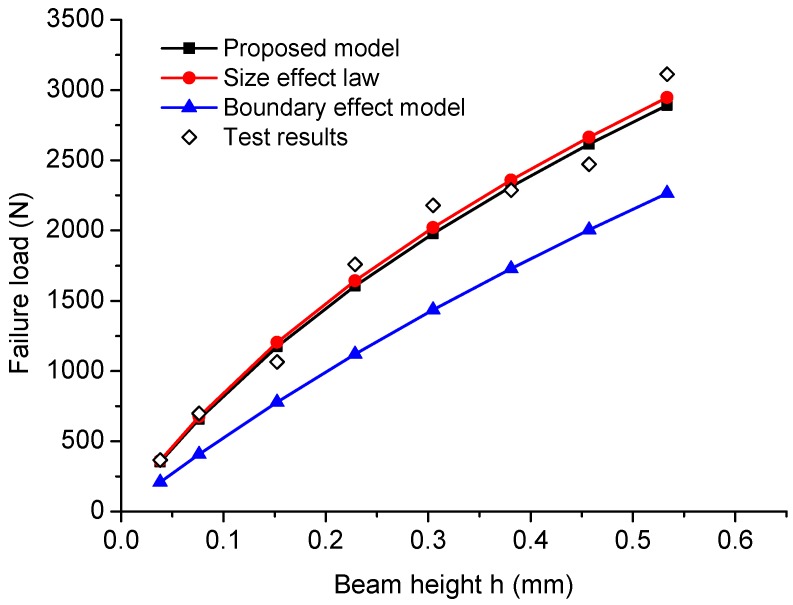
Model predictions of failure load versus beam height comparing with Type 2 SEL predictions, boundary effect model predictions and the test results of concrete.

With the geometrical information and mechanical parameters, the failure load $P_{\max}$ can be calculated by Equations (14) and (18). The predicted failure loads are plotted in Figure 10, showing good agreement with the test results and the predictions of Type 2 SEL. $\hat{B}f_{t}$ and $h_{0}$ in Type 2 SEL are obtained from the linear regression; therefore, the predictions of the Type 2 SEL deviate slightly from the predictions of the proposed model. However, if the length parameter $c_{f}$ in Type 2 SEL equals the transition crack length $a_{t}$ and the material tensile strength $f_{t}$ is known, then without any help of the experimental work, Type 2 SEL and the proposed model will give the same predictions of the failure loads. With the given material parameters, the boundary effect model gives predictions that are always smaller than the test results and the predictions of the proposed model and Type 2 SEL, which means the boundary effect model may underestimate the load bearing capacity of the cracked structure if the material parameters used in the model, including the tensile strength $f_{t}$ and fracture toughness $K_{c}$, are measured from the standard tests.

**Figure 11:**
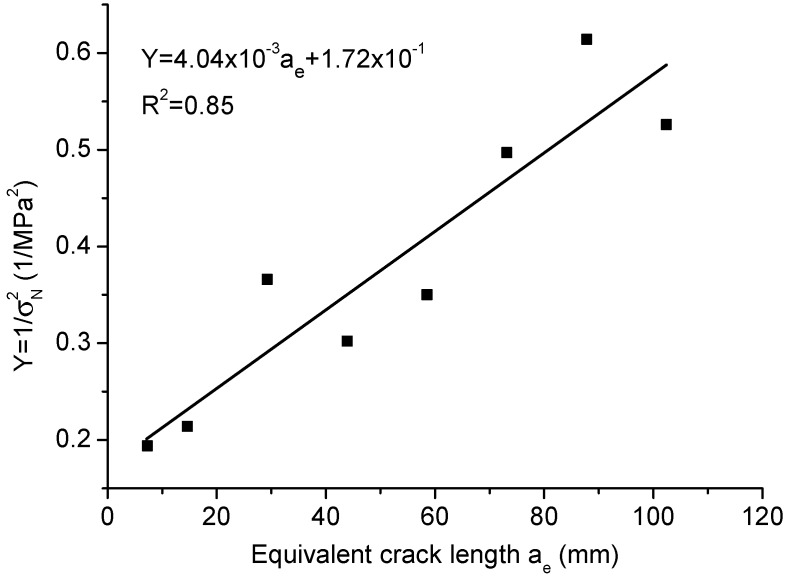
Linear regression on the test results of concrete.

For geometrically similar specimens, the mechanical properties can be identified from the test results. Figure 11 shows the fitted linear curve with slope $J={\left[H\left(\alpha \right)/\left(1.12f_{t}\right)\right]}^{2}/a_{t}=4.04\times 10^{-3}$ and intercept $C={\left[H\left(\alpha \right)/\left(1.12f_{t}\right)\right]}^{2}=1.72\times 10^{-1}$. The calculated transition crack length $a_{t}$ = 42.70 mm; tensile strength $f_{t}$ = 3.06 MPa; fracture toughness $K_{c}=\sqrt{G_{c}E}=1.21\;\mathrm{MPa}\cdot m^{1/2}$; which are all very close to the experimental measurements. For Type 2 SEL, the transitional height $h_{0}$ = 22.24 mm is obtained by the best fit, which contains the information of length parameter $c_{f}=h_{0}A^{2}\left(\alpha \right)\alpha /H^{2}\left(\alpha \right)$ = 42.70 mm. Since the measured transition crack length $a_{t}=42.66\;\mathrm{mm}\approx c_{f}$, the predictions given by the proposed model and Type 2 SEL are almost identical. In terms of the boundary effect model, in order to have the optimal fit for the test results, the fitted material parameters are $f_{t}$ = 5.42 MPa, $K_{c}=1.26\;\mathrm{MPa}\cdot m^{1/2}$ and $a_{t}$ = 13.61 mm. The fitted tensile strength is much higher than the direct measurement, which indicates that the predictions of the strength mechanism in the boundary effect model are inappropriate.

### 4.2. Limestone Experiments

Bažant et al. [34] tested four different sizes of C-TPB specimens made of Indiana limestone to investigate the size effect. The specimens have the same crack to height ratio α=0.4, the same span to height ratio S/h=4 and a fixed thickness *t* = 13 mm. The measured fracture toughness is $K_{c}=\sqrt{G_{c}E}=0.97\;\mathrm{MPa}\cdot m^{1/2}$. The tensile strength $f_{t}$ shows different values: Bažant obtained 3.45 MPa with the splitting tensile test; Jenq and Shah [35] got 5.0 MPa from the large double-edge cracked direct tensile test; and Schmidt obtained [36] 5.38MPa by six “direct pull” tests on “dog-bone specimens”.

The two geometrical correction factors A(α) and H(α) are 1.18 and 1.72, respectively. The beam dimensions, equivalent crack length $a_{e}$, failure loads $P_{\max}$ and the nominal strengths $\sigma_{N}$ are listed in Table 2.

**Figure 12:**
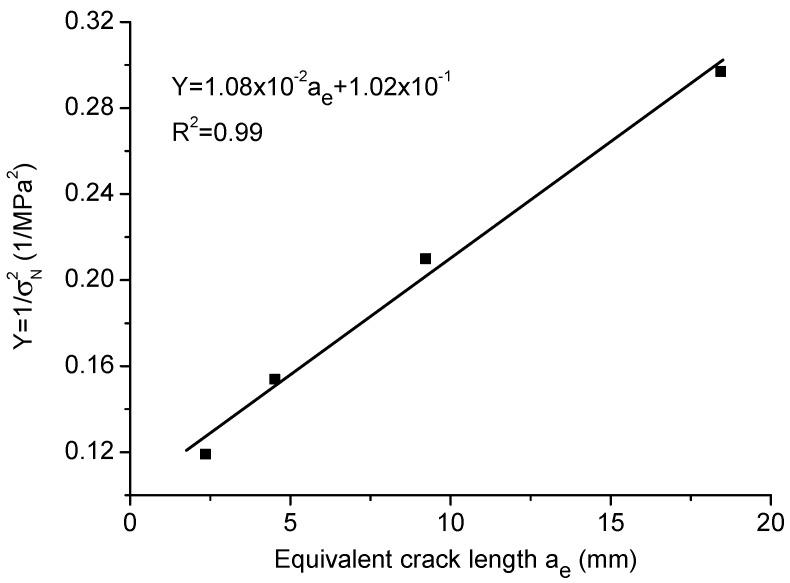
Linear regression on the test results of limestone.

In order to obtain the reasonable tensile strength $f_{t}$, the linear regression plot, as shown by the solid line in Figure 12, gives slope $J={\left[H\left(\alpha \right)/\left(1.12f_{t}\right)\right]}^{2}/a_{t}=1.08\times 10^{-2}$ and intercept $C={\left[H\left(\alpha \right)/\left(1.12f_{t}\right)\right]}^{2}=1.02\times 10^{-1}$. The calculated transition crack length $a_{t}$ = 9.39 mm; and the tensile strength $f_{t}$ = 4.91 MPa, which is inside the range of the measured results; the calculated fracture toughness $K_{c}=\sqrt{G_{c}E}=0.99\;\mathrm{MPa}\cdot m^{1/2}$, being very close to the experimental measurement of $0.97\;\mathrm{MPa}\cdot m^{1/2}$. For the boundary effect model, the fitted material parameters are $f_{t}$ = 8.72 MPa, $K_{c}=0.95\;\mathrm{MPa}\cdot m^{1/2}$ and $a_{t}$ = 29.83 mm, which are not close to nor inside the range of the measured values from the standard tests.

**Figure 13:**
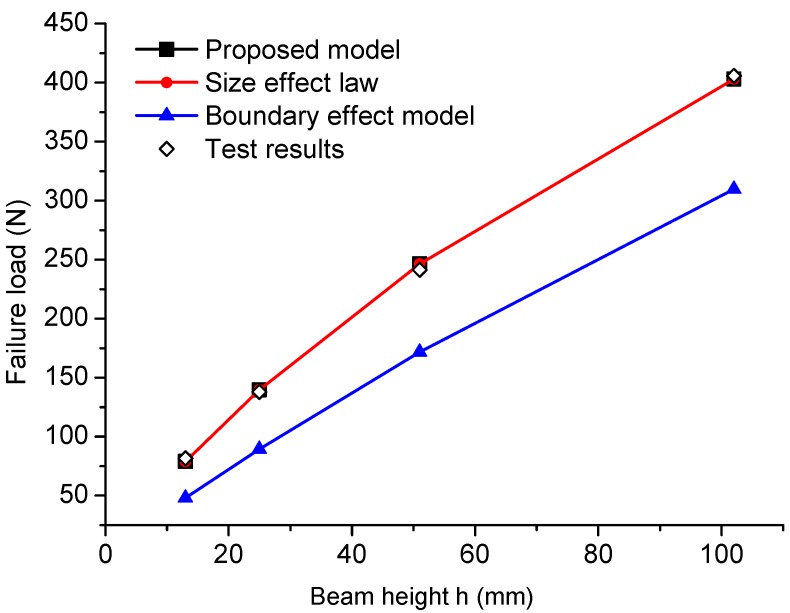
Model predictions of failure load versus beam height comparing with Type 2 SEL predictions, boundary effect model predictions and test results of limestone.

With the transition crack length $a_{t}$ and tensile strength $f_{t}$ obtained from the linear regression of the proposed model, the failure loads $P_{\max}$ then can be estimated and compared with the experimental results, the predictions of Type 2 SEL and the boundary effect model, as shown in Figure 13. It is not a surprise that the proposed model gives almost the same predictions as Type 2 SEL, because in Equation (17), the fitted $h_{t}$ and $\left(1.12f_{t}\right)/H\left(\alpha \right)$ are exactly the same as the $h_{0}$ and $\hat{B}f_{t}$ in Equation (2).

### 4.3. Hardened Cement Paste Experiments

**Figure 14:**
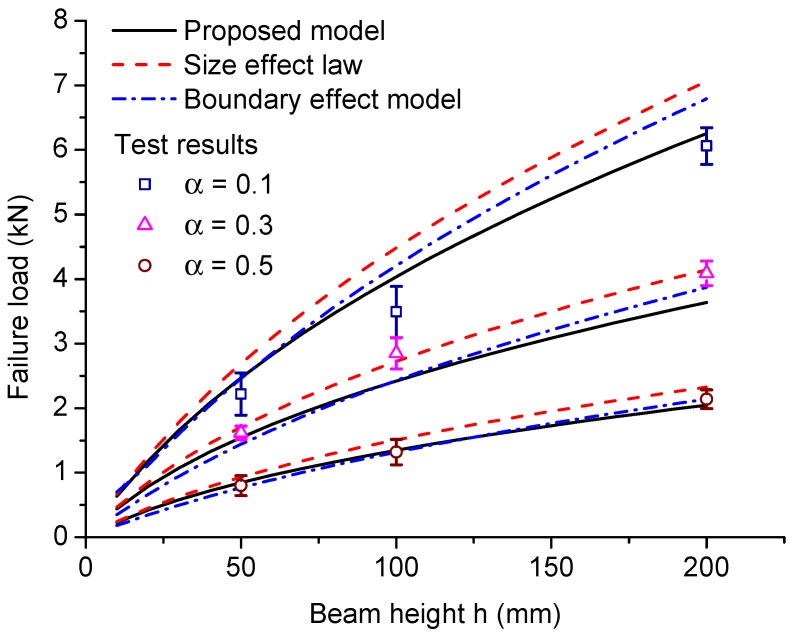
Model predictions of failure load versus beam height comparing with Type 2 SEL predictions, boundary effect model predictions and test results of hardened cement paste. The error bars indicate the standard deviations of the experimental results.

Karihaloo et al. [37] performed the C-TPB tests on cracked beams made of hardened cement paste with a span to height ratio of S/h=4. The heights of the beams are 50 mm, 100 mm and 200 mm, respectively, and the thickness *t* = 100 mm is fixed for all of the specimens. The crack to height ratios α are 0.1, 0.3 and 0.5. The hardened cement paste presents the following mechanical properties: averaged tensile strength $f_{t}$ = 3.53 MPa, Young’s modulus *E* = 20.8 GPa and fracture energy $G_{c}$ = 13.5 N/m, which is calculated by Equation (5) based on the measured failure load of the largest specimen. The corresponding transition crack length $a_{t}$ = 5.72 mm. The predicted failure loads given by the proposed model, Type 2 SEL and the boundary effect model and the test results are plotted in Figure 14, which shows that all of the models are capable of predicting acceptable results, but with different accuracies.

The Type 2 SEL parameters are provided by Yu et al. [25], which are calibrated for a/h=0.3. The fracture energy $G_{c}$ = 18.1 N/m, length scale $c_{f}$ = 7.2 mm, $\hat{B}f_{t}$ and transition size $h_{0}$ in Equation (2) are calculated by Equations (B1) and (B2) for crack to height ratios α=0.1, α=0.3 and α=0.5, respectively. For SEL, the results for small crack to height ratio α=0.1 are worse than the predictions of the proposed model and the boundary effect model; this is because the Type 2 SEL is not recommended for such small crack to height ratios, as it belongs to the transition of Type 1 SEL to Type 2 SEL. It should be noticed that the results for small crack to height ratios α≤0.1 should be properly fitted by the universal size effect law, which can describe this transition, but with much more complicated formulas [25,27]. In contrast to Type 2 SEL, The proposed model is easier to implement, and acceptable model predictions can be obtained for not only large crack to height ratios, but also for small ratios, for which Type 2 SEL is not recommended.

In terms of the boundary effect model, the material parameters $G_{c}$ = 18.2 N/m and $f_{t}$ = 4.58 MPa are obtained by the optimal fit. It is shown in Figure 14 that the proposed model works better than the boundary effect model for α=0.1. A smaller tensile strength $f_{t}\approx$ 3.50 MPa, which is almost equal to Karihaloo’s direct measurement, is required to fit the test results better for α=0.1. However, if this tensile strength is adopted, the predictions given by the boundary effect model for larger α values would deviate more from the test results than the predictions given by the proposed model and Type 2 SEL. The boundary effect model is easy to implement, the same as the proposed model, but a higher material tensile strength is required, for example 29.7% higher than the measured result for the hardened cement paste experiments studied in this section.

## 5. Conclusions

This article presents a new failure model to investigate the size effect and boundary effect in quasi-brittle materials. The model adopts the derivative of energy release rate $G^{\prime }$ to predict the failure of the strength mechanism and the energy criterion for the failure of the energy mechanism. An asymptotic model is developed to capture the effect of any crack size on the nominal strength $\sigma_{N}$, and its expression for geometrically similar specimens is also established, which is able to characterize the size effect induced by the specimen size.

The proposed model is compared with the boundary effect model and Type 2 SEL. The first expression (Equation (14)) of the proposed model is similar to the boundary effect model, which captures effectively the boundary effect. However, these two models are fundamentally different, due to the different assumptions for the strength mechanisms. An alternative expression (Equation (17)) of the proposed model for geometrically similar specimens is established and then compared with the Type 2 SEL, proving that when the length scale parameters in the proposed model and Type 2 SEL are identical, two models will give the same predictions. The advantage of the proposed model in contrast to Type 2 SEL is that the length parameter can be directly calculated from the measured material parameters of the standard tests, instead of being fitted from the geometrically similar tests. The model can be used for both geometrically similar specimens, as good as the Type 2 SEL, and the finite width specimen containing different crack sizes. What is more, the proposed model can give good predictions also for small crack to height ratios (α≤0.1), where Type 2 SEL is not recommended, and much more complicated formulas of USEL are required. Therefore, the scope of Type 2 SEL is somewhat extended. However, the proposed model is not a universal one like USEL, due to the fact that it cannot predict all of the size effects; for example, it cannot predict the size effect observed in un-cracked geometrically similar specimen tests, that is the crack initiation from the free surface.

In order to validate the proposed model, three sets of experimental results in the literature for limestone, for concrete and for hardened cement paste are used. It is shown that the predictions of the nominal strengths obtained from the proposed model are in very good agreement with the experimental results both for concrete and for limestone.

## Figures and Tables

**Table 1:** Concrete specimen and test results.

Specimen Dimensions (mm) [32]	$a_{e}$ (mm)	$P_{\max}\;\left(N\right)$ [32]	$\sigma_{N}$ (MPa)
152.4×38.1×25.4	7.32	366.53	2.27
304.8×76.2×25.4	14.63	721.28	2.24
609.6×152.4×25.4	29.27	1065.79	1.65
914.4×228.6×25.4	43.90	1759.72	1.82
1219.2×304.8×25.4	58.53	2179.63	1.69
1524×381×25.4	73.16	2288.61	1.42
1828.8×457.2×25.4	87.80	2470.99	1.28
2133.6×533.4×25.4	102.43	3113.76	1.38

**Table 2:** Limestone specimens and test results.

Specimen Dimensions (mm) [34]	$a_{e}$ (mm)	$P_{\max}\;\left(N\right)$ [34]	$\sigma_{N}$ (MPa)
52×13×13	2.35	78	2.77
		82	2.91
		85	3.02
100×25×13	4.52	134	2.47
		140	2.58
		140	2.58
204×51×13	9.22	238	2.15
		243	2.20
		243	2.20
408×102×13	18.44	394	1.78
		405	1.83
		418	1.89

**Figure A1:**
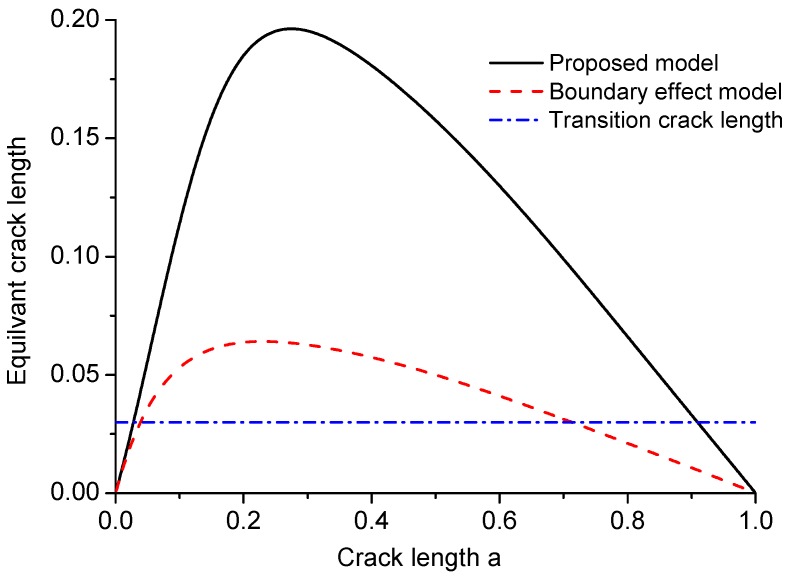
Equivalent crack length in the boundary effect model and the proposed model.

**Figure A2:**
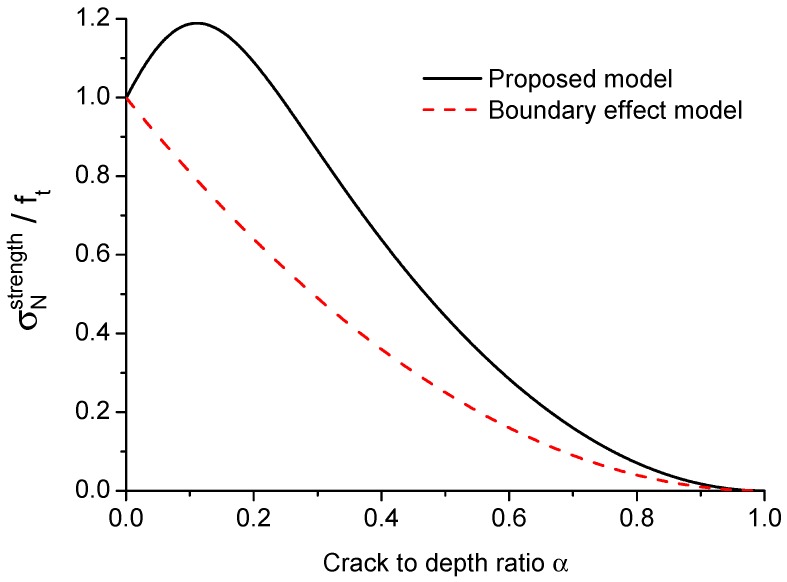
The ratio of the nominal strength predicted by the strength mechanism and tensile strength $\sigma_{N}^{\mathrm{strength}}/f_{t}$ for various crack to height ratios.

**Figure A3:**
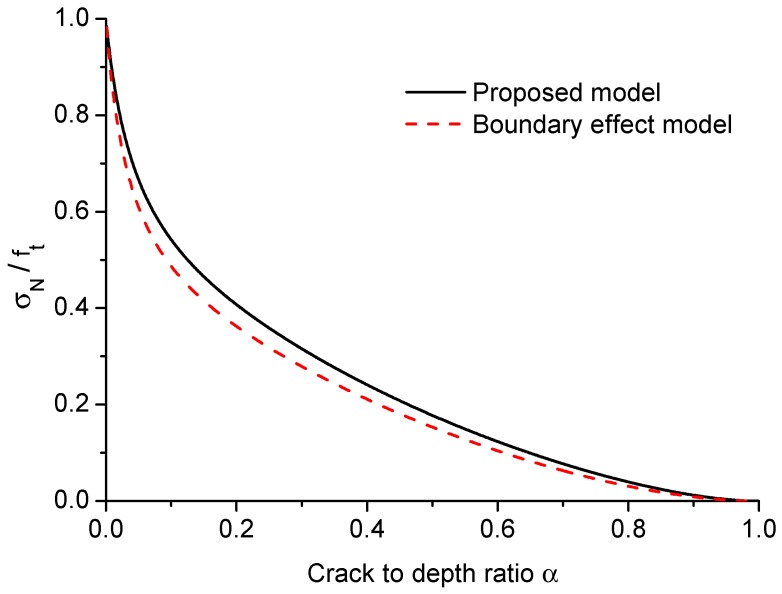
The ratio of nominal strength predicted by BEM and the proposed model and tensile strength $\sigma_{N}/f_{t}$ for various crack to height ratios.

**Figure A4:**
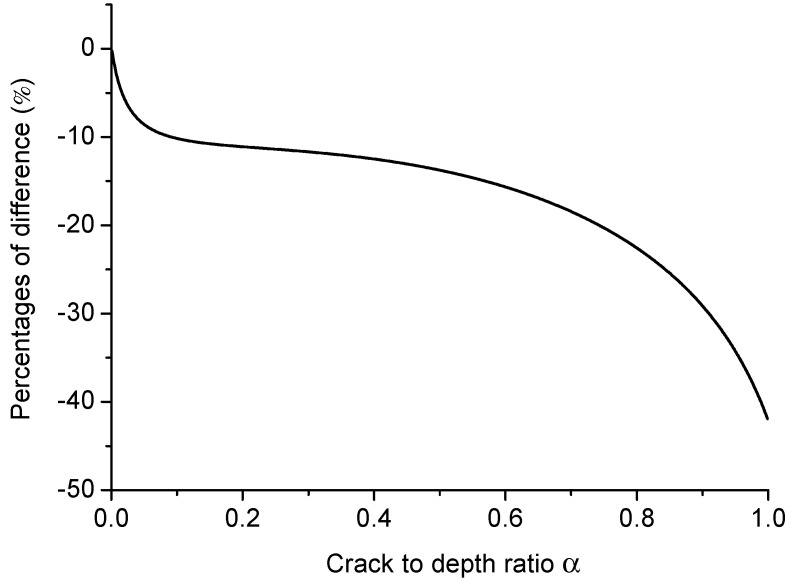
Percentages of the difference of the nominal strength given by the boundary effect model and the proposed model.

## Abbreviations

The following abbreviations are used in this manuscript:

BEM: Boundary Effect Model
C-TPB: Cracked Three-Point Bending
FPZ: Fracture Process Zone
LEFM: Linear Elastic Fracture Mechanics
SEL: Size Effect Law
SiC: Silicon Carbide
TCDs: Theories of Critical Distance
USEL: Universal Size Effect Law

## Appendix A. Comparison of the Proposed Model with the Hu–Duan Boundary Effect Model

According to boundary effect and using an equivalent crack length, Hu and Duan [6,7] proposed a Boundary Effect Model (BEM), which can be used to predict the nominal strengths of a finite or infinite width specimen containing different crack sizes. The boundary effect model for the rupture of a finite size specimen reads:

(A1) $$\sigma_{N}=B\left(\alpha \right)f_{t}{\left[1+\left(\frac{a_{e1}}{a_{t}}\right)\right]}^{-1/2}$$

where $B\left(\alpha \right)={\left(1-\alpha \right)}^{2}$ for the cracked three-point bending beam, $a_{e1}$ is referred to as the equivalent crack length in the boundary effect model and its value depends on the specimen geometry and crack length, which reads:

(A2) $$a_{e1}={\left[\frac{B(\alpha )\times A(\alpha )}{1.12}\right]}^{2}$$

Equations (A1) and (14) are crack size-based models, which estimate the effect of crack length on the nominal strength. Figure A1 shows the variation of equivalent crack length with respect to the real crack length *a* (beam height *h* = 1 m). For the same crack length, the equivalent crack length $a_{e}$ in the proposed model is higher than the definition of equivalent crack length $a_{e1}$ in the boundary effect model. Since the transition crack length $a_{t}$ shares the same definition in both models, the higher equivalent crack length $a_{e}$ (higher $a_{e}/a_{t}$) in the proposed model indicates that by using the proposed model, the rupture of the beam with a wider range of the crack length *a* will be dominated by the energy criterion.

The nominal strength given by the strength mechanism $\sigma_{N}^{\mathrm{strength}}$ in the boundary effect model is $B\left(\alpha \right)f_{t}$, while the proposed model believes that the strength mechanism is better characterized by the derivative of the energy release rate. Figure A2 presents the nominal strength predicted by the strength mechanism to tensile strength ratios $\sigma_{N}^{\mathrm{strength}}/f_{t}$, for various crack to height ratios α. The derivative of the energy release rate provides a higher nominal strength $\sigma_{N}^{\mathrm{strength}}$ than the boundary effect model for the same crack to height ratio α; while for the energy mechanism, both models give the same nominal strength $\sigma_{N}^{\mathrm{energy}}$, because a simple energy criterion is used in the two models.

The difference of the nominal strength given by the strength mechanism $\sigma_{N}^{\mathrm{strength}}$ will finally lead to the different nominal strengths predicted by the boundary effect model and the proposed model. Figure A3 shows the nominal strength predicted by the boundary effect model and the proposed model to tensile strength ratios $\sigma_{N}/f_{t}$, for various crack to height ratios, indicating that the proposed model gives higher nominal strength than the boundary effect model.

Figure A4 shows the percentages of the difference of the nominal strength given by the boundary effect model and the proposed model. With the increase of the crack length (fixed beam height), the difference of the nominal strength given by the two models will be larger and can reach more than 40% when α approaches one.

The same as the proposed model, BEM can also identify the material information from the test results. By setting $Y^{\prime }=1/\left(\sigma_{N}^{2}\right)$, Equation (A1) gives a linear regression plot $Y^{\prime }=J^{\prime }X^{\prime }+C^{\prime }$, with $X=a_{e1}$, $J^{\prime }=1/\left[a_{t}{\left(B\left(\alpha \right)f_{t}\right)}^{2}\right]$ and $C^{\prime }=1/[{\left(B\left(\alpha \right)f_{t}\right)}^{2}$. Hence, transition crack length $a_{t}=C^{\prime }/J^{\prime }$, material tensile strength $f_{t}=\sqrt{1/C^{\prime }}/B\left(\alpha \right)$ and fracture toughness $Kc=\left(1.12f_{t}\right)\sqrt{a_{t}\pi }$.

## Appendix B. Comparison of the Proposed Model with Type 2 SEL

In the expression of Type 2 SEL, $h_{0}$ is a constant proportional to Irwin’s characteristic length $l_{\mathrm{ch}}$, and $\hat{B}$ is a dimensionless constant characterizing the structure geometry. The expressions of these two values are given as follows [3]:

(B1) $$h_{0}=\frac{c_{f}g^{\prime }\left(\alpha \right)}{g(\alpha )}$$

(B2) $$\hat{B}f_{t}=\sqrt{\frac{EG_{c}}{g^{\prime }\left(\alpha \right)c_{f}}}$$

where $g\left(\alpha_{0}\right)=K_{I}^{2}\left(\alpha \right)h{\left(b/P\right)}^{2}=A^{2}\left(\alpha \right)\pi \alpha$ is the dimensionless energy release function of linear elastic fracture mechanics; $c_{f}$ is the effective size of the fracture process zone, which is proportional to the characteristic length $l_{\mathrm{ch}}$ and transition crack length $a_{t}$ and equals the total crack length, which gives the same (according to LEFM) specimen compliance as the actual crack with its process zone minus the initial crack length or traction free crack length $a_{0}$ [3], or approximately equals half length of FPZ [2]. Normally, $c_{f}$ is identified from the tests results of geometrically similar specimens, along with the transitional size $h_{0}$. It should be pointed out that when $c_{f}$ is known, Type 2 SEL can be applied to structures or specimens that are not geometrically similar.

Recall the definition of the correction factor for the derivative of energy release rate H(α) (Equation (8)); $g^{\prime }\left(\alpha_{0}\right)$ can be simplified as follows:

(B3) $$g^{\prime }\left(\alpha \right)=H^{2}\left(\alpha \right)\pi$$

After the substitution of Equations (4), (12), (B1)–(B3) into Equation (2) and some algebraic work, one gets:

(B4) $$\sigma_{N}=\frac{1.12f_{t}\sqrt{a_{t}/c_{f}}}{H(\alpha )}{\left(1+\frac{a_{e}}{c_{f}}\right)}^{-1/2}$$

When $c_{f}=a_{t}$, the proposed failure model shown in Equations (14) and (17) and Type 2 SEL are identical, although the proposed model is presented in two different forms. However, as a fitted parameter form of the test results, $c_{f}$ is not guaranteed to be equal to the transition crack length $a_{t}$ [26], which means two models will provide different nominal strength predictions when $c_{f}\neq a_{t}$. It should be noticed that $a_{t}$ can also be identified from the test results, instead of calculating from the material tensile strength $f_{t}$ and fracture energy $G_{c}$. In such cases, the proposed model and Type 2 SEL will provide the same nominal strengths, but the material properties ($f_{t}$ and $G_{c}$) fitted from the test results may be different.
